# Protective Mechanism of the Antioxidant Baicalein toward Hydroxyl Radical-Treated Bone Marrow-Derived Mesenchymal Stem Cells

**DOI:** 10.3390/molecules23010223

**Published:** 2018-01-20

**Authors:** Yage Tian, Xican Li, Hong Xie, Xiaozhen Wang, Yulu Xie, Chuanbing Chen, Dongfeng Chen

**Affiliations:** 1 School of Basic Medical Science, Guangzhou University of Chinese Medicine, Guangzhou 510006, China; rebecca-22222@163.com; 2 International Institute for Translational Chinese Medicine, Guangzhou University of Chinese Medicine, Guangzhou 510006, China; 3 School of Chinese Herbal Medicine, Guangzhou University of Chinese Medicine, Guangzhou 510006, China; xiehongxh1@163.com (H.X.); Jennywang2014@126.com (X.W.); xieyulu1900@163.com (Y.X.); henchuanbing@gzucm.edu.cn (C.C.); 4 Innovative Research & Development Laboratory of TCM, Guangzhou University of Chinese Medicine, Guangzhou 510006, China; 5 The Research Center of Basic Integrative Medicine, Guangzhou University of Chinese Medicine, Guangzhou 510006, China

**Keywords:** baicalein, 5,6,7-trihydroxyflavone, *e*-transfer, bmMSCs, Fe^2+^-chelating, antioxidant mechanism

## Abstract

Our study explores the antioxidant and cytoprotective effects of baicalein and further discusses the possible mechanisms. A methyl thiazolyl tetrazolium (MTT) assay revealed that baicalein could considerably enhance the viability of hydroxyl radical-treated bone marrow-mesenchymal stem cells (bmMSCs) at 37–370 µM. The highest viability rate was 120.4%. In subsequent studies, baicalein was observed to effectively scavenge hydroxyl radical and PTIO• radicals, reducing Fe^3+^ and Cu^2+^ ions. In the Fe^2+^-chelating UV-vis spectra, mixing of baicalein with Fe^2+^ yielded two evident redshifts (275 → 279 nm and 324 → 352 nm) and a broad absorption peak (λ_max_ ≈ 650 nm, ε = 1.6 × 10^3^ L mol^−1^·cm^−1^). Finally, we compared the Fe^2+^-chelating UV-vis spectra of baicalein and its analogues, including 5-hydroxyflavone, 6-hydroxyflavone, 7-hydroxyflavone, catechol, pyrogallol, and chrysin. This analysis revealed that the 4-keto group of the C-ring played a role. The 5,6,7-trihydroxy-group (pyrogallol group) in the A-ring served as an auxochrome, enhancing the absorbance of the UV-vis spectra and deepening the color of the Fe^2+^-complex. We concluded that baicalein, as an effective hydroxyl radical-scavenger, can protect bmMSCs from hydroxyl radical-mediated oxidative stress. Its hydroxyl radical-scavenging effects are likely exerted via two pathways: direct scavenging of hydroxyl radicals, possibly through electron transfer, and indirect inhibition of hydroxyl radical generation via Fe^2+^ chelation through the 4-keto-5,6,7-trihydroxy groups.

## 1. Introduction

Hydrogen peroxide (H_2_O_2_) can cross cell membranes freely and are often found in biological systems [1]. When mixed with ferrous iron (Fe^2+^), it will undergo the Fenton reaction (Fe^2+^ + H_2_O_2_ → Fe^3+^ + •OH + HO^−^), generating a hydroxyl radical (•OH), well-known as the most harmful reactive oxygen species (ROS). Hydroxyl radicals can attack all types of cellular biomolecules (especially DNA) to cause oxidative stress [2,3].

Oxidative stress not only lowers viability of bone marrow-derived mesenchymal stem cells (bmMSCs), but also induces their differentiation [3,4]. It is reported that mild oxidative stress can induce differentiation of bmMSCs to adipose cells, but not to osteose or nerve cells [5,6]. Thus, iron overload, a cause of •OH radical generation, can usually lead to bone loss [7]. These aforementioned detrimental effects of oxidative stress currently limit the clinical application of bmMSC transplantation for neurodegenerative diseases (e.g., Parkinson’s disease) [8] and bone diseases (e.g., osteoporosis) [9,10]. As such, scientists are searching for an effective antioxidant from natural products [11] or synthetic compounds [12,13] to relieve •OH radical-mediated oxidative stress, to improve bmMSCs viability enough for the clinical application [14].

**Figure 1:**
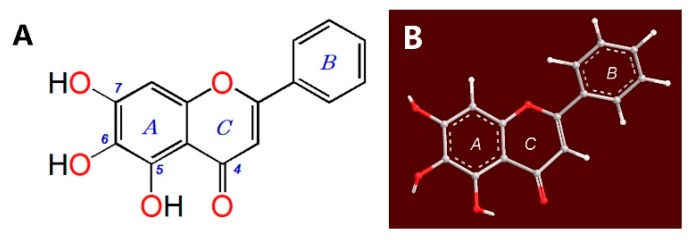
Structure (**A**) and preferential conformation-based ball-and-stick model (**B**) of baicalein (5,6,7-trihydroxyflavone).

Baicalein (Figure 1), a natural flavonoid occurring in traditional Chinese herbal medicine, *Scutellaria baicalensis* Georgi, is predicted to be useful as an antioxidant. Recently, baicalein has been demonstrated to suppress the early stages of adipogenesis [15,16] and regulate bone formation [17]. In addition, baicalein has been reported to protect HS-SY5Y cells from hydrogen peroxide-induced oxidative stress [18] and to attenuate neurological injury in rats [19].

Nevertheless, there is no direct evidence for the beneficial effects of baicalein toward •OH-treated bmMSCs. This study therefore used a methyl thiazolyl tetrazolium (MTT) assay to assess its protective effects towards •OH-treated bmMSCs, thus providing information for the use of baicalein in bmMSC transplantation technology. 

More importantly, there are some disputes regarding the antioxidation mechanisms (especially Fe^2+^-chelation) of baicalein. Yoshino and colleagues have suggested that baicalein could inhibit •OH radical generation [20], while Shieh and colleagues have reported that baicalein could not scavenge •OH [21]. Regarding Fe^2+^-chelation chemistry, Ren and colleagues stated that the 4-keto group of flavonoids plays a critical role in this process [22], while Perez and colleagues argued that Fe^2+^-chelation mainly occurred at the 6,7-dihydroxyl groups in flavonoids [23]. Thus, our study used various chemical approaches to explore the possible antioxidation mechanisms, especially the Fe^2+^-chelation pathway. We believe that this study will help settle the above disputes. 

## 2. Results and Discussion

**Figure 2:**
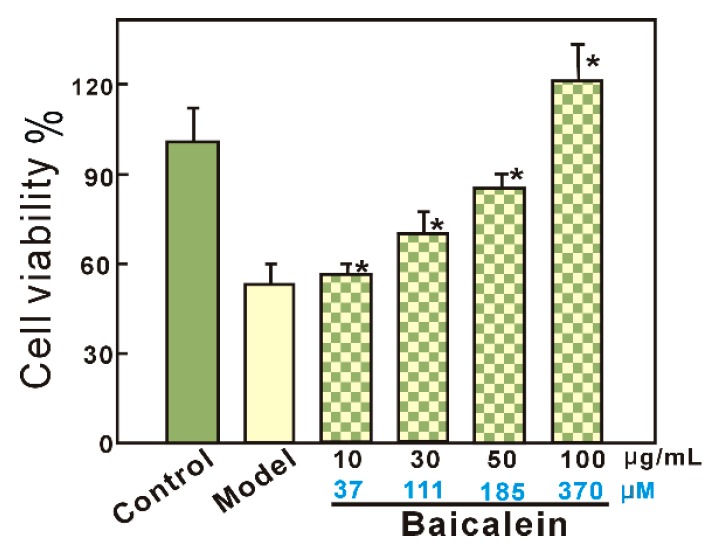
Protective effect of baicalein toward •OH-treated bmMSCs. Cell viability was assessed using the MTT method. •OH radicals were generated by addition of FeCl_2_ (100 μM) followed by H_2_O_2_ (50 μM). The control group was cultured in medium only, while the model group was treated with •OH radicals. The baicalein group was treated by •OH followed by baicalein. Each value is expressed as the mean ± SD, *n* = 3; * Significant difference vs. model group, *p* < 0.05. bmMSCs, bone marrow-derived mesenchymal stem cells; MTT, methyl thiazolyl tetrazolium.

As seen in Figure 2, in the model group, bmMSCs damaged by •OH radicals showed only 52.9 ± 12.0% viability. However, when damaged bmMSCs were further treated with baicalein at 10–100 μg/mL (37–370 μM), the viability was completely restored, and further increased to 120.7 ± 4.3% (baicalein group). This result suggests that baicalein protects •OH radical-treated bmMSCs, as supported by previous studies [24,25].

**Figure 3:**
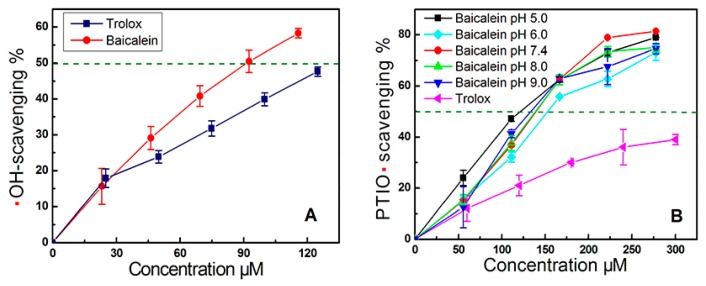
Dose-response curves of baicalein in the •OH-scavenging assay (**A**) and in PTIO•-scavenging assay (**B**). PTIO•-scavenging assay was conducted at pH 5.0, 6.0, 7.4, 8.0, and 9.0. Trolox served as positive control. Each value is expressed as the mean ± SD, *n* = 3.

The damage to the cells may be largely due to •OH attack, as •OH radicals are much more toxic than H_2_O_2_ alone. Correspondingly, the above cytoprotective effects of baicalein are attributable to its •OH-scavenging ability [26,27]. In fact, in the •OH-scavenging assay based on deoxyribose degradation, baicalein was observed to have an effective dose-dependent response (Figure 3A). The IC_50, Trolox_/IC_50, Baicalein_ value (1.46) in Table 1 suggests that baicalein is a stronger •OH-scavenger than the standard antioxidant, Trolox. Our data appear to support the opinions of Yoshino [20], and disagree with those of Shieh [21].

Since •OH generation relies on Fe^2+^ catalysis of the Fenton reaction, attenuating Fe^2+^ levels via a chelation pathway can indirectly inhibit the production of •OH radicals in cells [28]. This is sometimes called indirect •OH-inhibition. Correspondingly, direct scavenging of •OH-radicals that were already generated is known as direct •OH-scavenging [29]. However, it is impossible to verify whether •OH radicals are directly scavenged, due to the transience of the •OH radical (half-life of 10^−9^ s). We therefore used a stable oxygen-centered PTIO• radical for the exploration. As seen in Figure 3B, baicalein scavenged PTIO• radicals in a dose-dependent manner at various pH values. These data suggest that baicalein may scavenge •OH directly.

**Figure 4:**
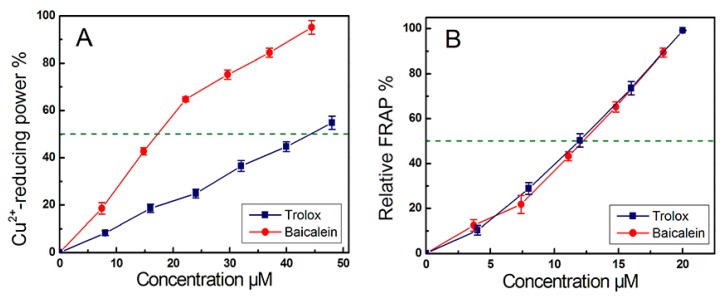
Dose-response curves of baicalein in Cu^2+^-reducing (**A**) and Fe^3+^-reducing (FRAP, **B**) assays (Values are expressed as the mean ± SD, *n* = 3).

The evidence from cyclic voltammetry suggested that at a pH ≤ 5.0, PTIO• could be scavenged via an electron transfer (ET) pathway [30]. At pH 5.0, baicalein could scavenge PTIO• in a concentration-dependent manner (Figure 3B), suggesting that baicalein exerts its effect possibly via ET. This was further supported by our Cu^2+^-reducing and Fe^3+^-reducing (FRAP) assays. As illustrated in Figure 4, baicalein, in addition to the positive control Trolox, increased the relative Cu^2+^-reducing and Fe^3+^-reducing percentages, in a concentration-dependent manner. In particular, the FRAP assay demonstrated the presence of an ET reaction, because in acidic solution (below pH 3.6), a high concentration of H^+^ successfully suppresses H^+^ ionization from a phenolic antioxidant (i.e., baicalein) [31]. The above Fe^3+^-reducing reaction of baicalein, can therefore be viewed as merely an ET process. Recently, Marino and colleagues used density functional theory to predict that during the process of •OH-scavenging of gallic acid (a phenolic antioxidant), an ET reaction was involved [32]. All of this experimental and theoretical evidence suggests that an ET reaction may involve the direct •OH-scavenging process of baicalein. 

As mentioned in our previous report [33], Fe^3+^-reduction by an antioxidant may form a new recycle for supplying Fe^2+^ ions. This is regarded as being a cause of pro-oxidation [34]. In fact, as a flavonoid, baicalein has been reported to possess pro-oxidation potential, by Huang and colleagues [35]. Yoshino and Murakami have argued that baicalein could enhance the oxidation of Fe^2+^ to block this pro-oxidation mechanism [20]. These contradictory findings may be due to the differences in flavonoid concentrations [36]. Nevertheless, in the present study, no pro-oxidation potential was observed in cellular assays (Figure 2), or in antioxidant assays in vitro (Figure 3A). 

**Figure 5:**
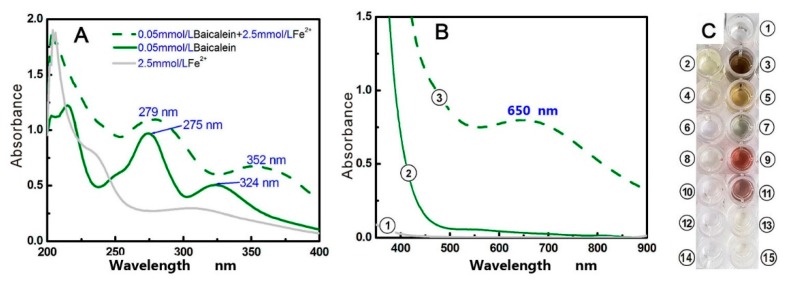
(**A**) UV spectra of a low-concentration of baicalein and a baicalein-Fe^2+^ complex; (**B**) vis spectra of high-concentration baicalein and the baicalein-Fe^2+^ complex; (**C**) colors of the various solutions. (**①** 25 mM Fe^2+^; **②** 0.5 mM baicalein; **③** 0.5 mM baicalein + 25 mM Fe^2+^; **④** 0.5 mM pyrogallol; **⑤** 0.5 mM pyrogallol + 25 mM Fe^2+^; **⑥** 0.5 mM catechol; **⑦** 0.5 mM catechol + 25 mM Fe^2+^; **⑧** 0.5 mM chrysin; **⑨** 0.5 mM chrysin + 25 mM Fe^2+^; **⑩** 0.5 mM 5-hydroxyflavone; **⑪** 0.5 mM 5-hydroxyflavone + 25 mM Fe^2+^; **⑫** 0.5 mM 6-hydroxyflavone; **⑬** 0.5 mM 6-hydroxyflavone + 25 mM Fe^2+^; **⑭** 0.5 mM 7-hydroxyflavone; and **⑮** 0.5 mM 7-hydroxyflavone + 25 mM Fe^2+^. The UV spectra of a low concentration of chrysin, 5-hydroxyflavone, pyrogallol, and chrysin-Fe^2+^ complex are detailed in Supplementary 2. The vis spectra of **④**–**⑮** are detailed in Supplementary 1).

In addition to direct •OH-scavenging, an indirect •OH-inhibition pathway (i.e., Fe^2+^-chelation) was also investigated in our study. As seen in Figure 5A,B, after mixing with Fe^2+^ solutions, baicalein presented stronger UV peaks and a darker green color, suggesting that Fe^2+^-chelation may occur and that baicalein may indirectly inhibit •OH radical production via an Fe^2+^-chelation pathway. However, there is a dispute regarding the Fe^2+^-chelating site and the role of the 4-keto group, as mentioned above. To address this problem, we selected six analogues for comparative study; 5-hydroxyflavone, 6-hydroxyflavone, 7-hydroxyflavone, catechol, pyrogallol, and chrysin. 

As seen in Supplementary 1 and Figure 5, when mixed with Fe^2+^, neither the 6-hydroxyflavone solution nor the 7-hydroxyflavone solution developed color, and gave corresponding peaks in the visible spectra. Our results agree with previously published literature reporting that the isolated hydroxyl-groups cannot chelate Fe^2+^ [37]. In comparison, when a hydroxyl-group is adjacent to another hydroxyl-group, the situation may be different. As seen in Figure 5 and Supplementary 1, *ortho*-dihydroxy groups (i.e., catechol groups) or adjacent 1,2,3-trihydroxy-groups (i.e., pyrogallol group), can chelate Fe^2+^ to produce a light color and weaker visible spectra. This indicates that Fe^2+^-chelation requires at least two adjacent dihydroxy groups. This is consistent with previous reports that flavones with catechol group have a similar Fe^2+^-chelating capacity to those with pyrogallol group [27,37,38].

When a hydroxyl-group is adjacent to a keto group, it can possess Fe^2+^-chelating potential. As seen in Supplementary 1 and Figure 5, 5-hydroxyflavone could also chelate Fe^2+^ to yield a dark color and an evident visible spectra peak (λ_max_ = 557 nm, ε = 1.4 × 10^3^ L mol^−1^·cm^−1^). This result clearly suggests that adjacent hydroxyl-keto groups (i.e., 4-keto-5-hydroxyl) can chelate Fe^2+^. Chrysin, with a 4-keto-5-hydroxyl group, could also chelate Fe^2+^ to produce a darker orange color and stronger UV-vis spectra (λ_max_ = 528 nm, ε = 1.5 × 10^3^ L mol^−1^·cm^−1^). We therefore conclude that adjacent hydroxyl-keto groups possess Fe^2+^-chelating potential. This phenomenon is similar to the copper chelation of flavonol, which is reported to have the two most efficient copper chelation sites (i.e., the 3-hydroxy-4-keto group and the 5,6,7-trihydroxy-group) [39]. 

Importantly, the spectra of hydroxyl-groups differ from those of keto groups. A keto group attached to an aromatic nucleus can act as a chromophore, while a phenolic hydroxyl-group can act only as an auxochrome group. Therefore, adjacent hydroxyl-keto groups can generate darker colors and stronger visible spectra peaks than those produced by catechol groups or pyrogallol groups. It is clear that if a 4-keto group is adjacent to a hydroxyl-group in flavonoids, the 4-keto group actually plays a critical role in Fe^2+^-chelation reactions. Our data undoubtedly support the findings of Ren [22] and several other researchers [40,41] and are contradictory to the opinions of Perez [23]. 

**Figure 6:**
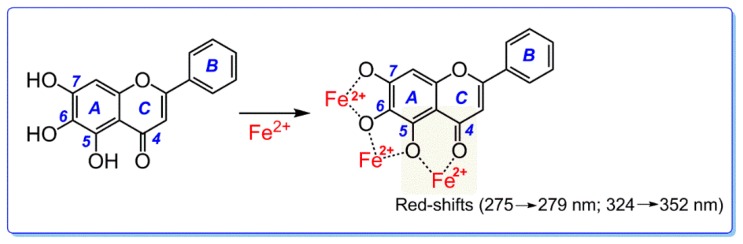
The proposed Fe^2+^-chelation reaction of baicalein (including UV-vis spectra assignments).

Based on the above discussion, we deduce that baicalein actually possesses three Fe^2+^-chelating sites; between the 5,6-dihydroxyl groups, between the 6,7-dihydroxyl groups, and between the 4-keto-5-hydroxy groups. The preferential conformation-based ball-and-stick model indicates that the huge 4-keto-5,6,7-trihydroxy groups have a planar configuration (Figure 1B and Supplementary 3), and thus, the Fe^2+^-chelating complex is stable. The proposed Fe^2+^-chelation reaction of baicalein is shown in Figure 6 [27,37]. 

As seen in Figure 5A, baicalein generated two red shifts (275 nm → 279 nm and 324 nm → 352 nm) in the UV spectra bands. The fact that neither catechol nor pyrogallol without 4-keto exhibited similar peaks, while chrysin with 4-keto gave similar peaks, implies that the two redshifts can thus be attributed to the Fe^2+^-chelation reaction at the 4-keto-5-hydroxy group (Supplementary 2).

In the visible spectra, baicalein produced a broader, stronger peak (approximately 650 nm) than any other analogue (Figure 5B). The broad, strong peak is also partly from Fe^2+^-chelation at the 4-keto-5-hydroxy group. From the perspective of spectroscopy, the three phenolic hydroxyl-groups at the 5, 6, and 7-positions, as auxochrome groups, can enhance absorbance of UV-vis spectra peak. In other words, the broad, strong peak near 650 nm can be regarded as the overlying of UV-vis spectra from the above three Fe^2+^-chelating sites. Correspondingly, baicalein also generated the darkest color in the Fe^2+^-chelation reaction, compared to the six analogues. 

**Figure 7:**
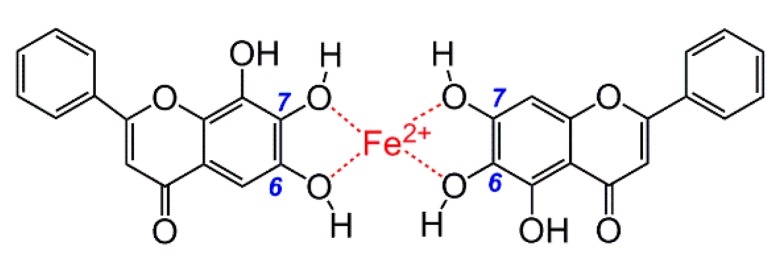
The structure allowing baicalein molecules to jointly chelate one Fe^2+^ ion [23].

It must be noted that (1) despite many reports on the metal-chelating ability of flavonoids [37,38,39,40,41,42] and descriptions of Na^+^(Al^3+^) interacting with flavonoids [43], no study has clearly indicated the roles of specific groups within flavonoids; (2) The 4-keto group has been disregarded because the flavonoid levels exceeded the Fe^2+^ level in the previous experiment. In this case, two baicalein molecules are able to jointly chelate one Fe^2+^ ion (Figure 7) [23]. However, even with the intake of sufficient flavanone-enriched juices or foods, flavonoids levels (1.75 ± 0.35 μM [44], 2.16 μM, and 3.47 μM [45]) are still much lower than Fe^2+^ levels (34.95 ± 13.35 μM [46]) in human plasma. Thus, the experimental result that 4-keto plays a negligible role in Fe^2+^-chelation of flavonoids [23] lacks biological relevance.

Taken together, when baicalein chelates Fe^2+^ ions in a biological system, the 4-keto group of the C-ring actually plays a key role, similar to a chromophore. Of course, the 4-keto must be adjacent a hydroxy-group (i.e., the site between 4-keto and 5-hydroxy groups). Fe^2+^-chelation can also occur in the 5,6,7-trihydroxy-groups (i.e., the site between the 5,6-dihydroxyl groups and the site between 6,7-dihydroxyl groups). However, such Fe^2+^-chelation at the 5,6,7-trihydroxy-group plays a minor role, similar to an auxochrome; it can enhance the Fe^2+^-chelating UV-vis spectra absorbance and Fe^2+^-complex color. Thus, the Fe^2+^-chelation reaction of baicalein occurs in 4-keto-5,6,7-trihydroxy groups, among which 4-keto groups play a key role.

## 3. Materials and Methods

### 3.1. Chemicals and Animals 

Baicalein (CAS number: 491-67-8, 98%) and chrysin (CAS number: 480-40-0, 98%) were obtained from Chengdu Biopurify Phytochemicals, Ltd. (Chengdu, China, Supplementary 4); 5-Hydroxylflavone (CAS number: 491-78-1, 98%), 6-hydroxylflavone (CAS number: 6665-83-4, 98%), 7-hydroxylflavone (CAS number: 6665-86-7, 98%), and 2-phenyl-4,4,5,5-tetramethylimidazoline-1-oxyl-3-oxide radical (PTIO•) were from TCI Development Co., Ltd. (Shanghai, China); (±)-6-Hydroxyl-2,5,7,8-tetramethlychromane-2-carboxylic acid (Trolox), 2,9-dimethyl-1,10-phenanthroline (neocuproine), 2,4,6-tripyridyltriazine (TPTZ), catechol, pyrogallol, and 3-(4,5-dimethylthiazol-2-yl)-2,5-diphenyltetrazolium bromide (MTT) were from Sigma-Aldrich Shanghai Trading Co. (Shanghai, China). Deoxyribose was obtained from Amresco, Inc. (Solon, OH, USA). Dulbecco’s modified Eagle’s medium (DMEM) and fetal bovine serum (FBS) were purchased from Gibco (Grand Island, NY, USA); CD44 and Proteinase K were purchased from Wuhan Boster Co., Ltd. (Wuhan, China). All other reagents were of analytical grade. Sprague–Dawley (SD) rats 4 weeks of age were obtained from the animal center of the Guangzhou University of Chinese Medicine. The protocol of this experiment was performed under the supervision of the Institutional Animal Ethics Committee at the Guangzhou University of Chinese Medicine.

### 3.2. Protective Effect against •OH-Induced Damage to bmMSCs (MTT Assay)

**Figure 8:**
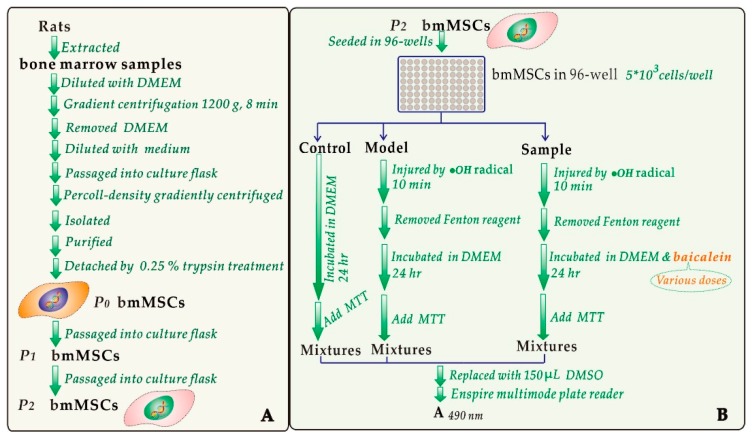
Experimental procedures for the preparation and culture of bmMSCs (**A**) and for the MTT assay (**B**).

The bmMSCs were prepared by our laboratory from four-week-old SD rats. The experimental procedures were based on our previous study [47] and are shown in Figure 8A. The resulting bmMSCs were evaluated for the purity by flow cytometry. Only bmMSCs with 95–97% purity could be further used for the MTT assay to evaluate the cytoprotective effect of baicalein (Figure 8B) [3,27]. 

(Enspire multimode plate reader was the product of Perkin Elmer Singapore Pte. Ltd., Singapore). Each test was repeated in five independent wells. MTT was used at 5 mg/mL (in PBS), and the addition volume was 20 μL. The addition of Fenton reagent was conducted by injection of FeCl_2_ (100 μM) followed by H_2_O_2_ (50 μM).

### 3.3. Hydroxyl Radical (•OH) Scavenging Assay 

The •OH-scavenging activity was investigated using our method [48]. In brief, all test samples were dissolved in ethanol (1 mg/mL), and a 10–50 μL sample solution was transferred to mini tubes; the ethanol solvent was then removed at 80 °C to eliminate its interference. The reactions were performed in 0.2 M phosphate buffer (pH 7.4) containing 2.8 mM deoxyribose, 2.8 mM H_2_O_2_, 25 μM FeCl_3_ 80 μM Na_2_EDTA, and the test sample (10–50 μg). The reaction was started by adding ascorbic acid to a final concentration of 100 μM, and the reaction mixture (600 μL in total) was incubated for 20 min at 50 °C in a water bath. After incubation, the color was developed by adding 0.5 mL of 2-thiobarbituric acid (1 g/100 mL) followed by 0.5 mL of trichloroacetic acid (5 g/100 mL) and heating the sample in a boiling water bath for 15 min. The sample was cooled and diluted twofold with 95% ethanol, and the absorbance was measured at 532 nm against buffer (as blank). The reaction mixture not containing test sample was used as a control. The scavenging activity on hydroxyl radicals was expressed as

(1) $$Inhibition\%=\frac{A_{0}-A}{A_{0}}\times 100\%$$

where *A*_0_ is the absorbance of the control without sample and *A* is the absorbance of the reaction mixture with sample. 

### 3.4. PTIO• Scavenging Assay

The PTIO• scavenging assay was also based on our method [49]. In the PTIO• scavenging assay, 80 μL of an aqueous PTIO• solution (0.1 mM) was mixed with 20 μL of phosphate buffer (pH 5.0, 6.0, 7.4, 8.0, and 9.0) with the sample at various concentrations. The mixture was maintained at 37 °C for 30 min, and the absorbance at 560 nm was measured using a microplate reader (Multiskan FC, Thermo Scientific, Shanghai, China). The PTIO• inhibition percentage was calculated using the formula described in Section 3.3.

### 3.5. Cu^2+^-Reducing Power Assay

The cupric ion (Cu^2+^) reducing power capacity was based on a published method [50] with slight modification. Briefly, 250 μL of a CuSO_4_ aqueous solution (10 mM), 250 μL of a neocuproine ethanolic solution (7.5 mM) and 250 μL of a CH_3_COONH_4_ buffer solution (100 mM, pH 7.0) were added to a test tube containing baicalein (2–12 μL). The total volume was adjusted with the buffer to 1 mL, and the solution was mixed vigorously. The absorbance compared to a buffer blank was measured at 450 nm after 30 min. Increased absorbance of neocuproine-Cu^+^ complex in the reaction mixture indicates increased reduction capability. Trolox was used as a positive control. The percentage reducing power of the sample compared to the maximum absorbance tested in baicalein at 12 μg/mL was calculated based on the following formula:

(2) $$Relative\;reducing\;effect\%=\frac{A-A_{\min}}{A_{\max}-A_{\min}}\times 100\%$$

where *A*_min_ is the absorbance of the control without sample, *A* is the absorbance of the reaction mixture with sample, and *A*_max_ is the greatest absorbance of the reaction mixture with sample.

### 3.6. Ferric (Fe^3+^) Reducing Antioxidant Power (FRAP) Assay

The FRAP assay was adapted from Benzie and Strain [51]. Briefly, the FRAP reagent was freshly prepared by mixing 10 mM TPTZ, 20 mM FeCl_3_ and 0.25 M pH 3.6 acetate buffer at 1:1:10 (volume ratio). The test sample (*x* = 1–5 μL, 0.1 mg/mL) was added to (20 − *x*) μL of 95% ethanol followed by 80 μL of FRAP reagent. The absorbance was measured at 595 nm after a 30-min incubation at ambient temperatures using distilled water as the blank. The relative reducing power of the sample compared to the maximum absorbance was calculated by the formula presented in Section 3.5.

### 3.7. Ultraviolet-Visible (UV-Vis) Spectra Determination of Fe^2+^ Binding

UV-vis spectral determination was conducted according to the published method [52,53] with minor modifications. In brief, 260 μL of a methanolic solution of baicalein (1 mg/mL) and 400 μL of an aqueous solution of FeCl_2_·4H_2_O (25 mg/mL) were added to 1340 μL of methanol. The solution was mixed vigorously and incubated at room temperature for 30 min. Subsequently, the product mixture was collected, and a spectrum was obtained from 200 to 900 nm using a UV-Vis spectrophotometer (Unico 2600A, Shanghai, China). Next, 200 μL of the supernatant was transferred to a 96-well plate and imaged using a smartphone (Huawei, Honor 8, Shenzhen, China).

### 3.8. Statistical Analysis

Each experiment was performed in triplicate, and the data were recorded as the mean ± SD (standard deviation). The IC_50_ value was defined as the final concentration causing 50% radical inhibition (or relative reducing power). Statistical comparisons were made by one-way ANOVA to detect significant differences using SPSS 13.0 (SPSS Inc., Chicago, IL, USA) for Windows. *p* < 0.05 was considered to be statistically significant.

## 4. Conclusions

Baicalein, as an effective hydroxyl radical-scavenger, can protect bmMSCs from hydroxyl radical-induced oxidative stress. Baicalein scavenges hydroxyl radicals through two pathways: direct scavenging of hydroxyl radicals via an ET pathway, and indirect inhibition of hydroxyl radical production via an Fe^2+^-chelation pathway, which occurs in 4-keto-5,6,7-trihydroxy groups.

## Figures and Tables

**Table 1:** IC_50_ values of baicalein and Trolox in various assays.

Assays	Baicalein μM	Trolox μM	Ratio value IC_50, Trolox_/IC_50, Baicalein_
•OH-scavenging	93.7 ± 1.6 ^a^	137.6 ± 3.6 ^b^	1.46
PTIO• scavenging *	188.7 ± 13.1 ^a^	384.2 ± 23.3 ^b^	2.03
Cu^2+^ reducing	19.0 ± 0.1 ^a^	44.6 ± 1.5 ^b^	2.34
FRAP	11.1 ± 0.0 ^a^	12.0 ± 0.0 ^a^	0.96

IC_50_ value was defined as the final concentration of 50% radical-scavenging (relative reducing power), was calculated by linear regression analysis, and is expressed as the mean ± SD (*n* = 3). Linear regression was analyzed using Origin 6.0 professional software. The mean values with different superscripts (a or b) in the same row, are significantly different (*p* < 0.05). * The assay was conducted at pH 7.4. The ratio value is defined as IC_50, Trolox_/IC_50, Baicalein_. FRAP, Fe^3+^ reducing antioxidant power assay. PTIO•, 2-phenyl-4,4,5,5-tetramethylimidazoline-1-oxyl-3-oxide radical.

## Supplementary Materials

Supplementary materials are available online, include: 1. The visible spectra of **④**–**⑮**. 2. The UV spectra of low-concentration chrysin and chrysin-Fe^2+^ complex. 3. Preferential conformation-based ball-and-stick model of baicalein-Fe^2+^ complex. 4. Information about origin of baicalein and chrysin.

## Abbreviations

ABTS	2,2′-azino-bis (3-ethylbenzo-thiazoline-6-sulfonic acid)
bmMSCs	bone marrow-derived mesenchymal stem cells
DMEM	Dulbecco’s modified Eagle’s medium
ET	Electron transfer
FBS	Fetal bovine serum
FRAP	ferric ion reducing antioxidant power
MTT	methyl thiazolyl tetrazolium
Neocuproine	2,9-dimethyl-1,10-phenanthroline
PTIO•	2-phenyl-4,4,5,5-tetramethylimidazoline-1-oxyl-3-oxide radical
ROS	reactive oxygen species
Trolox	(±)-6-hydroxyl-2,5,7,8-tetramethlychromane-2-carboxylic acid
TPTZ	2,4,6-tripyridyl triazine
