# Supplementary material for: Protective Mechanism of the Antioxidant Baicalein toward Hydroxyl Radical-Treated Bone Marrow-Derived Mesenchymal Stem Cells
Source: Molecules. 2018 Jan 20;23(1):223. doi: 10.3390/molecules23010223 (PMC6017293; doi:10.3390/molecules23010223)
Supplement: Supplementary file 1 [file molecules-23-00223-s001.zip › Suppl.s/Suppl. 4- Certificates.pdf]

成都普瑞法科技开发有限公司
Chengdu Biopurify Phytochemicals Ltd.

文件编码（DOC NO.）：PRF-COA-BP023201

产品分析证书
Certificate of Analysis

中文名称：黄芩素

English Name：Baicalein
别名**(Alias)**: 5,6,7-Trihydroxyflavone; Noroxylin

产品编码**(Cat. No.)**:BP0232
CAS Number: 491-67-8
分子式**(M. F.)**: C15H10O5
分子量**(M. W.)**: 270.24

批号**(Batch No.)**: PRF8020642
报告日期**(Report date)**: 2017年2月6日

Structure

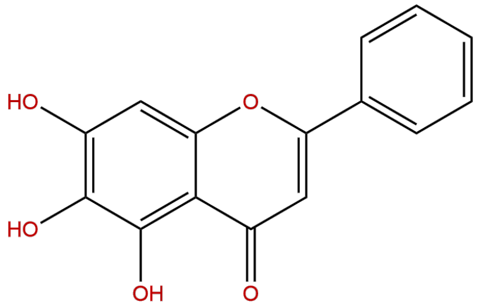

检验结果（**Analytical result**）：

检验项目（**Test Item**）	检验指标（**Specifications**）	检验结果（**Results**）
外观Appearance	Yellow powder	Yellow powder
纯度Purity (HPLC-DAD, 275nm)*	≥98.0%	99.30%
质谱Mass	270.2±1	Conforms
核磁NMR	Comply with the structure	Conforms
水分Water Content	＜3.0%	0.9%

*** 色谱图见附件（Please find HPLC chromatography attached.）**

贮存条件（Storage）:2~8℃, protected from light, keep package airproofed when not in use.

复测期（Retest date）:two years (2019-02-05) under conditions list above.

QC: Zhang Ling
Date: 2017年2月6日

Chengdu Biopurify Phytochemicals Ltd. 成都普瑞法科技开发有限公司 质检专用章 Quality Approval Seal

QA: Wu Qi
Date: 2017年2月6日

备注**(Remarks)**: The sample solutions should be prepared and used on the same day, it is the best preparing the solutions immediately before use. If the solutons have to be made up in advance, it should be made as aliquots in tightly sealed vials at less than -20℃. Generally, these might be useable for up to two weeks.

In case of quality issue, please contact us within 15 days after receipt of the product.

Tel：+86-28-82633397　　Fax：+86-28-82633165

http://www.phytopurify.com　　Email：sales@biopurify.com　　biopurify@gmail.com

成都普瑞法科技开发有限公司
Chengdu Biopurify Phytochemicals Ltd.

Add: No.11 Building, No. 388 Rongtaidadao CNSTP
Wenjiang Zone, Chengdu, Sichuan, 611130 China
TEL: 028-82633987 FAX: 028-82633165
E-mail: biopurify@gmail.com sales@biopurify.com
Web: www.biopurify.com

Certificate of Analysis

Structure:

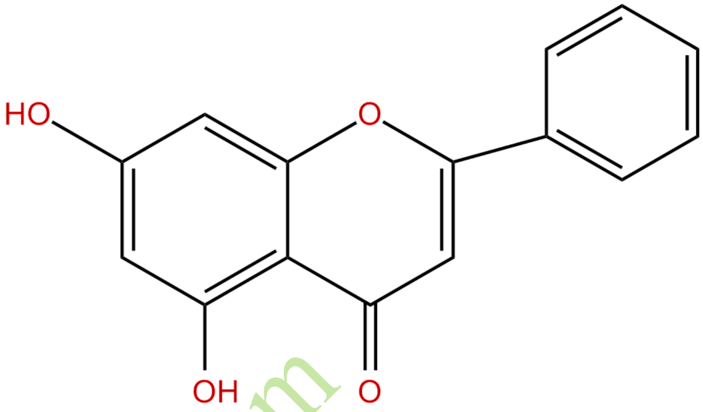

Product Name: Chrysin

Other Name: Chrysinic acid

Catalogue No.: BP0346

Batch No.: PRF7080712

Report date: 2016-08-07

CAS Number: 480-40-0

Mol. Formula: $C_{15}H_{10}O_4$

Mol. Weight: 254.24

Botanical Source: Ulmus sieboldiana

Type of compound:

Identification Method: Mass, NMR

Analysis Method of Purity: HPLC-DAD

www.biopurify.com

Analytical result:

**Test**	**Specification**	**Results**
Appearance	Yellow Powder	Yellow Powder
Loss on drying	＜3.0%	1.9%
Purity (HPLC,268 nm)*	≥98.0%	99.77%

***** Please find HPLC chromatography attached.

Package: Brown vial or HDPE Plastic Bottle

Storage: Cool and Dry place, protected from light, keep package airproofed when not in use.

Expiration: two years (2018-08-07) under conditions list above.

QC: Meng Pan

Date: 2016-08-07

QA: Lianglei Zhang

Date: 2016-08-07

成都普瑞法科技开发有限公司
Chengdu Biopurify Phytochemicals Ltd.

Empower 2 software

Chengdu Biopurify Phytochemicals Ltd

SAMPLE INFORMATION

Sample Name:	Chrysin PRF7080712	Acquired By:	panmeng
Sample Type:	Unknown	Sample Set Name:	
Vial:	1:E,7	Acq. Method Set:	Chrysin
Injection #:	1	Processing Method:	Samples
Injection Volume:	10.00 ul	Channel Name:	268.0nm
Run Time:	20.0 Minutes	Proc. Chnl. Descr.:	PDA 268.0 nm
Date Acquired:	2016-8-7 16:43:53 CST		
Date Processed:	2016-8-7 17:06:13 CST		

Auto-Scaled Chromatogram

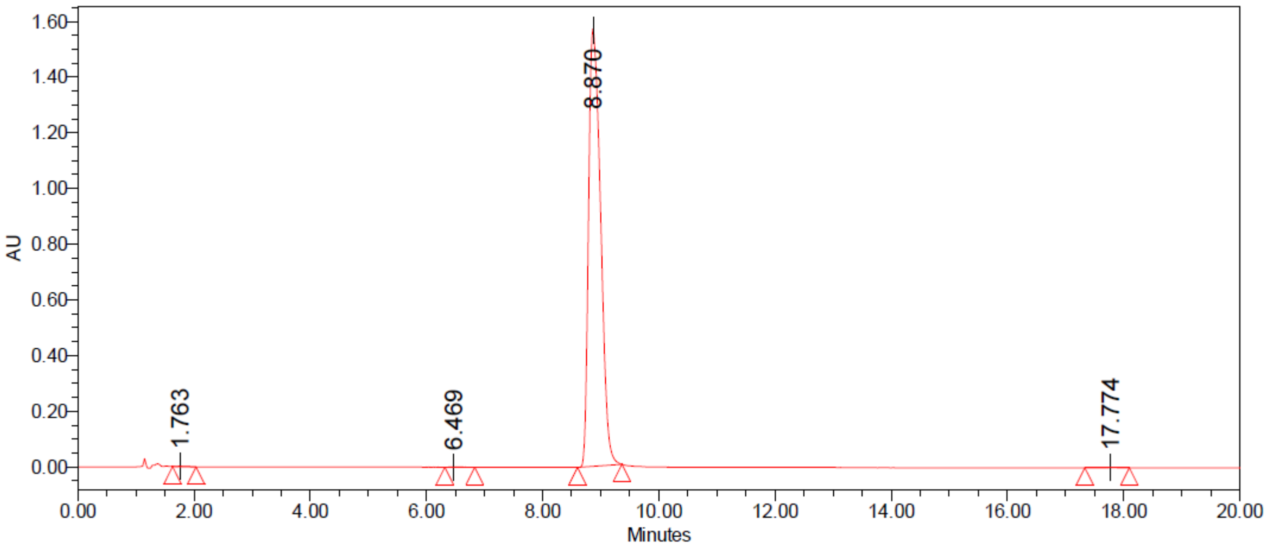

Peak Results

	Name	RT	Area	% Area	USP Plate Count	USP Resolution
1		1.763	20040	0.09	2371.72	
2		6.469	15698	0.07	4882.74	6.31
3		8.870	22526451	99.77	8681.83	7.16
4		17.774	15678	0.07	30397.12	9.46

EM=270

8020642-LCMS Scan ESI+

Ret. Time: 3.80

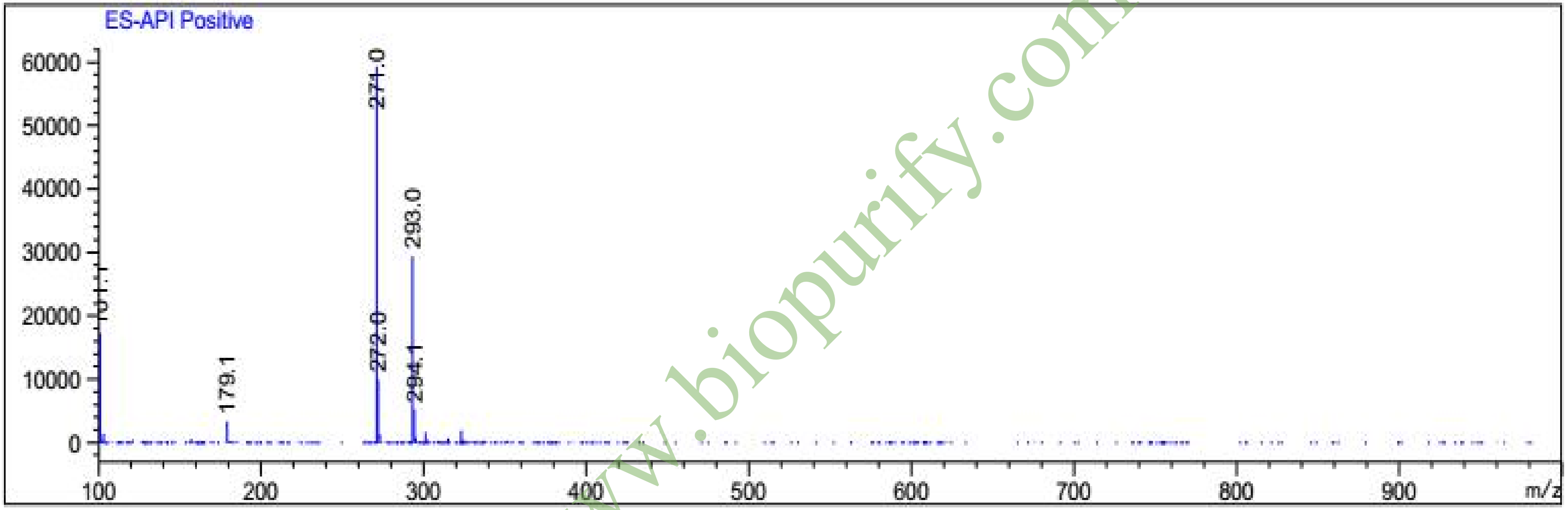

www.biopurify.com

EM=270

8020642-LCMS Scan ESI-

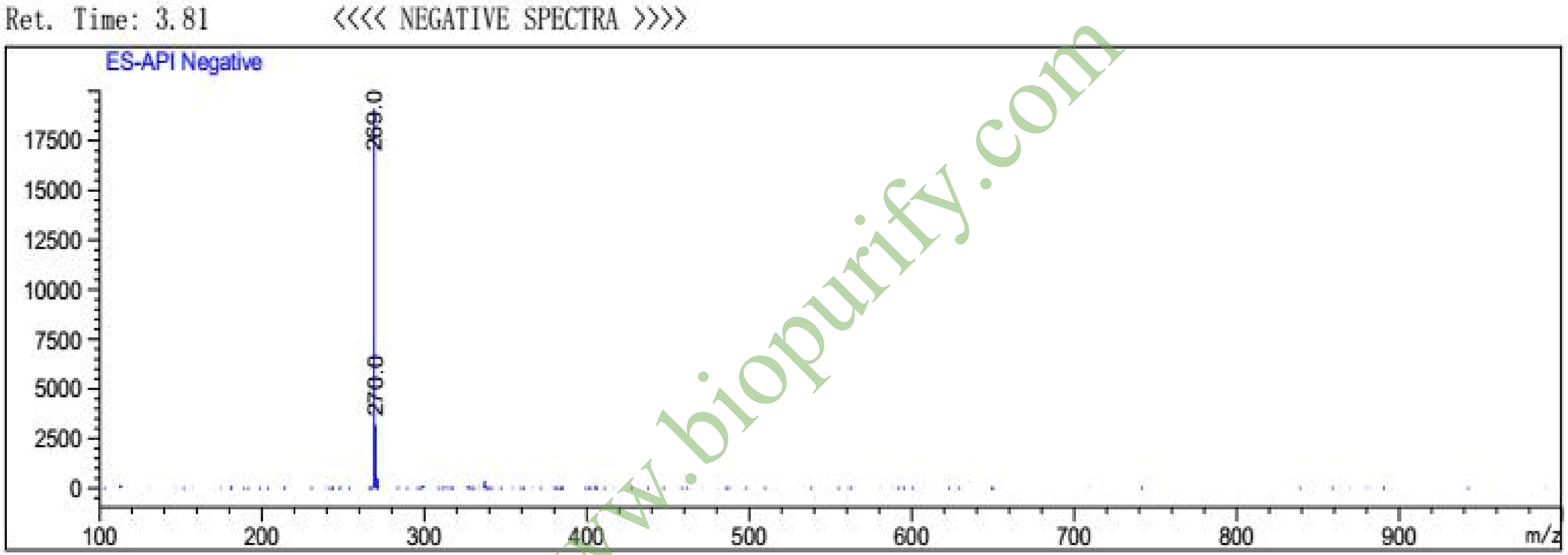

www.biopurify.com

Thermo Fisher Nicolet 6700	溴化钾压片
Baicalein 8020642	测试人：L

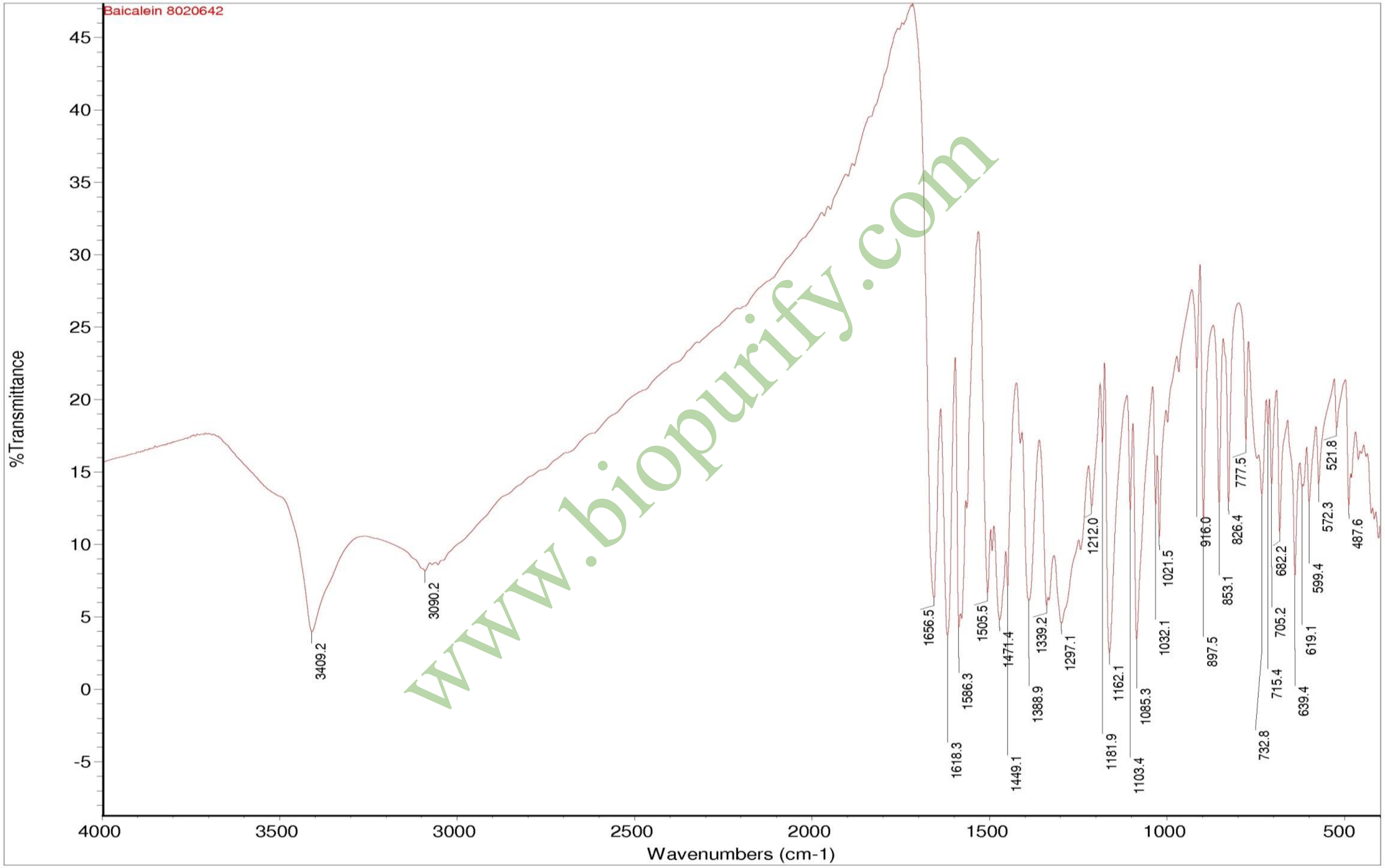

www.biopurify.com

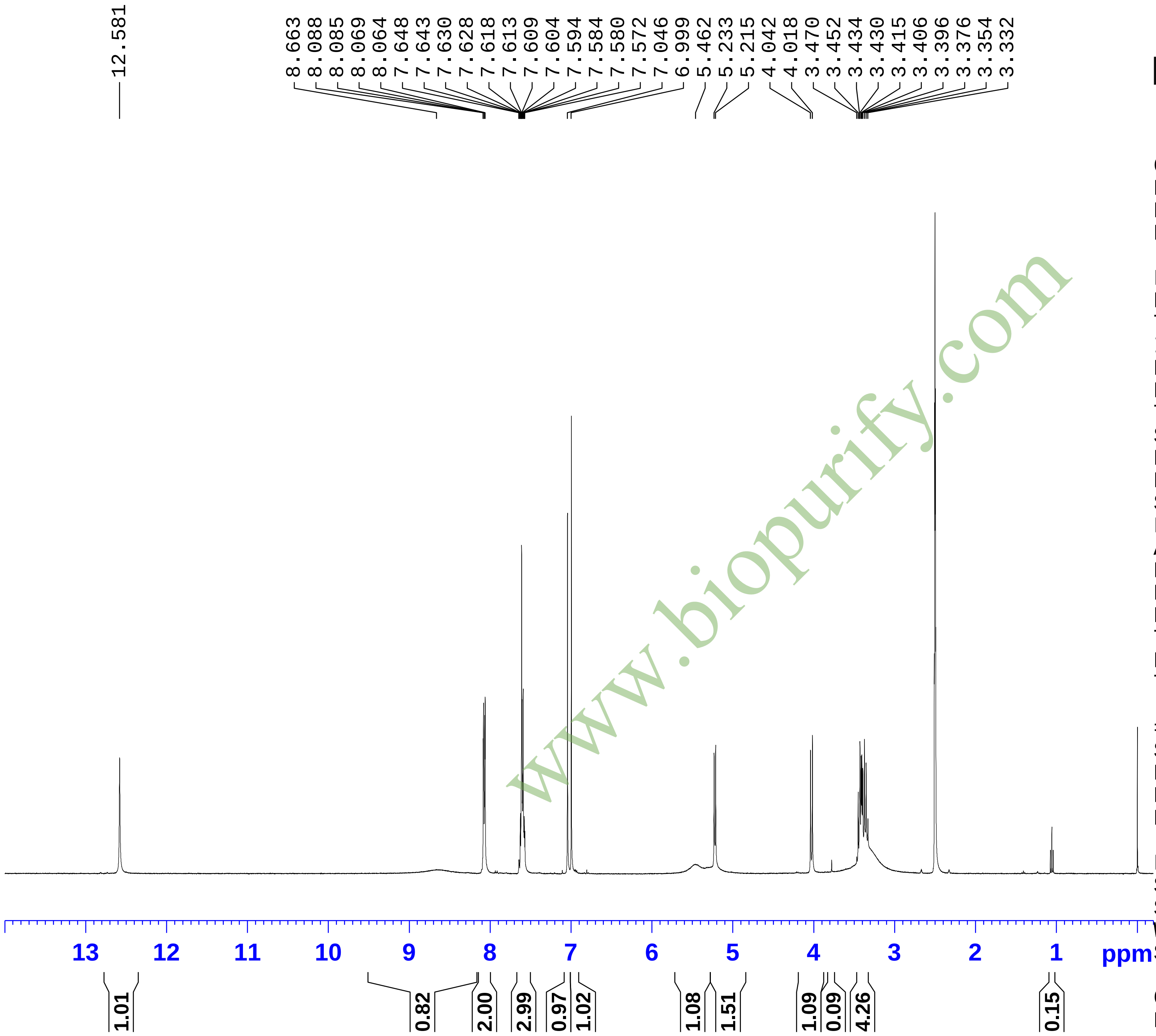

www.biopurify.com

Current Data Parameters
NAME Baicalin-1H NMR DMSO
EXPNO 1
PROCNO 1

F2 - Acquisition Parameters
Date_ 20170225
Time 17.16
INSTRUM spect
PROBHD 5 mm PABBO BB/
PULPROG zg30
TD 65536
SOLVENT DMSO
NS 16
DS 2
SWH 8012.820 Hz
FIDRES 0.122266 Hz
AQ 4.0894465 sec
RG 202.5
DW 62.400 usec
DE 6.50 usec
TE 301.2 K
D1 2.00000000 sec
TD0 1

======== CHANNEL f1 ========
SFO1 400.1324710 MHz
NUC1 1H
P1 14.79 usec
PLW1 11.50800037 W

F2 - Processing parameters
SI 65536
SF 400.1300023 MHz
WDW EM
SSB 0
LB 0.30 Hz
GB 0
PC 1.00
SR 2.34 Hz

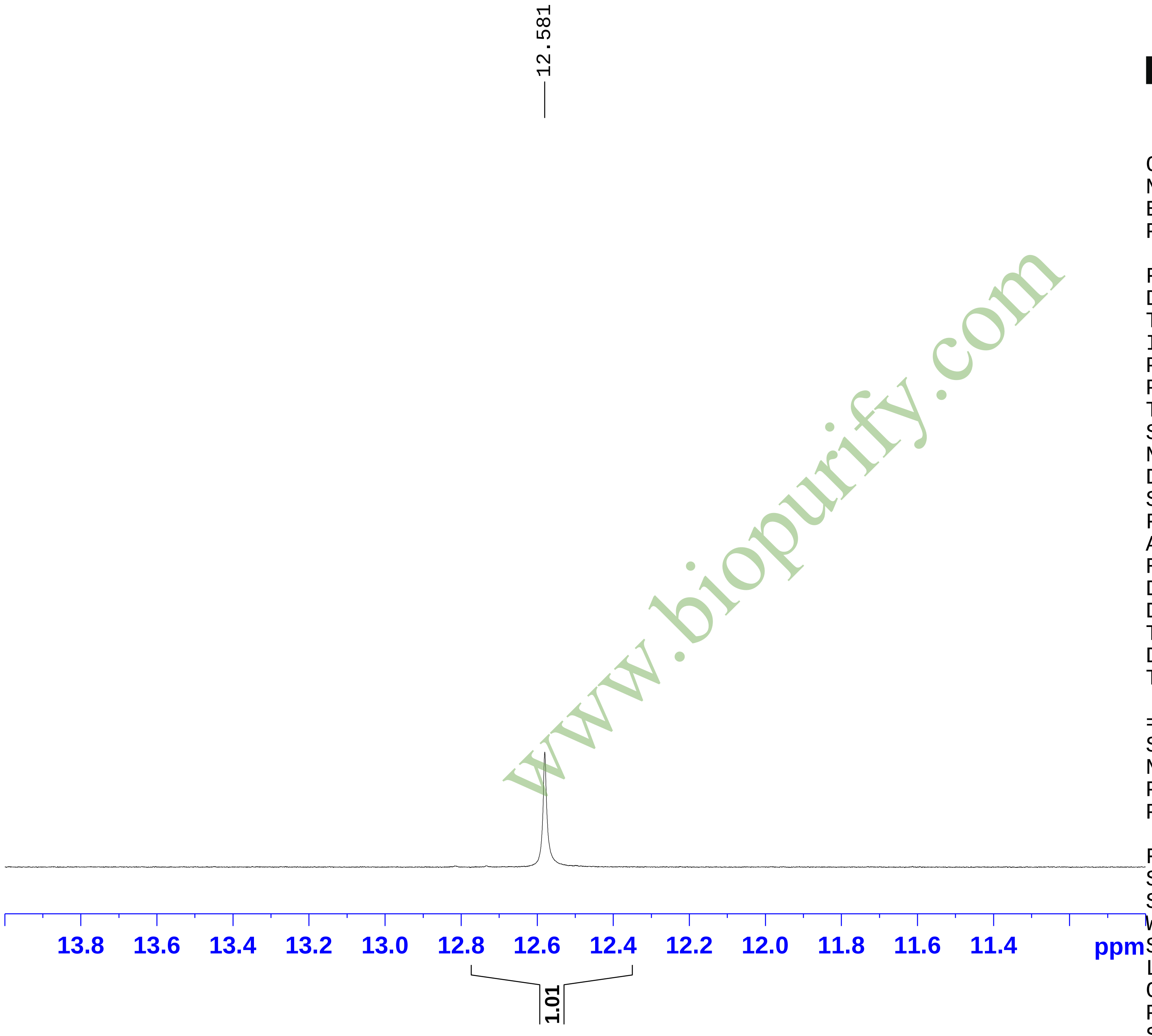

www.biopurify.com

BRUKER

Current Data Parameters
NAME Baicalin-1H NMR DMSO
EXPNO 1
PROCNO 1

F2 - Acquisition Parameters
Date_ 20170225
Time 17.16
INSTRUM spect
PROBHD 5 mm PABBO BB/
PULPROG zg30
TD 65536
SOLVENT DMSO
NS 16
DS 2
SWH 8012.820 Hz
FIDRES 0.122266 Hz
AQ 4.0894465 sec
RG 202.5
DW 62.400 usec
DE 6.50 usec
TE 301.2 K
D1 2.00000000 sec
TD0 1

======== CHANNEL f1 ========
SFO1 400.1324710 MHz
NUC1 1H
P1 14.79 usec
PLW1 11.50800037 W

F2 - Processing parameters
SI 65536
SF 400.1300023 MHz
WDW EM
SSB 0
LB 0.30 Hz
GB 0
PC 1.00
SR 2.34 Hz

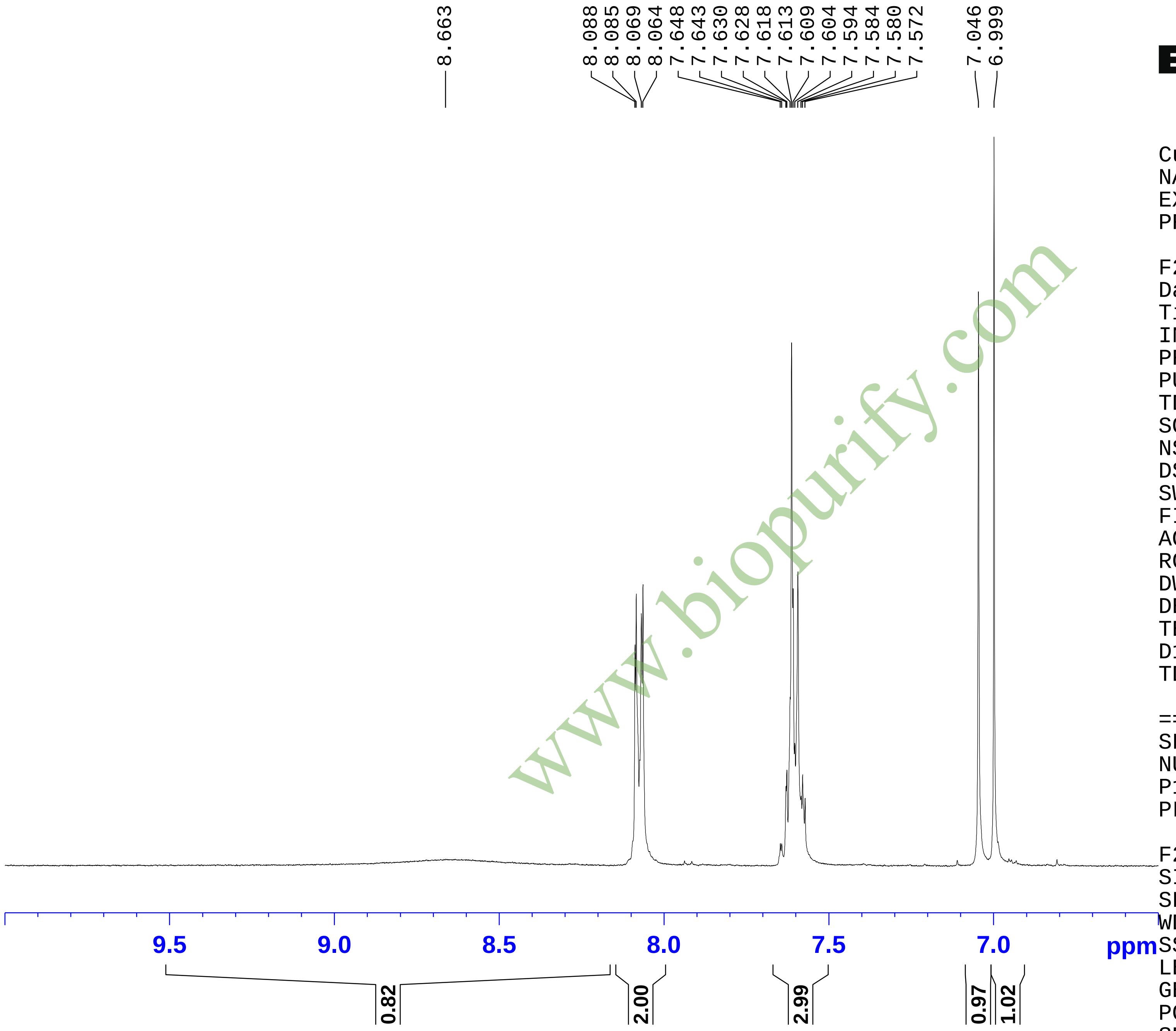

www.biopurify.com

```
Current Data Parameters
NAME      Baicalin-1H NMR DMSO
EXPNO                     1
PROCNO                    1

F2 - Acquisition Parameters
Date_              20170225
Time                  17.16
INSTRUM               spect
PROBHD     5 mm PABBO BB/
PULPROG                zg30
TD                    65536
SOLVENT                DMSO
NS                       16
DS                        2
SWH            8012.820 Hz
FIDRES         0.122266 Hz
AQ            4.0894465 sec
RG                    202.5
DW               62.400 usec
DE                 6.50 usec
TE                301.2 K
D1           2.00000000 sec
TD0                       1

======== CHANNEL f1 ========
SFO1        400.1324710 MHz
NUC1                     1H
P1                14.79 usec
PLW1        11.50800037 W

F2 - Processing parameters
SI                    65536
SF          400.1300023 MHz
WDW                      EM
SSB       0
LB                 0.30 Hz
GB        0
PC                     1.00
SR                 2.34 Hz
```

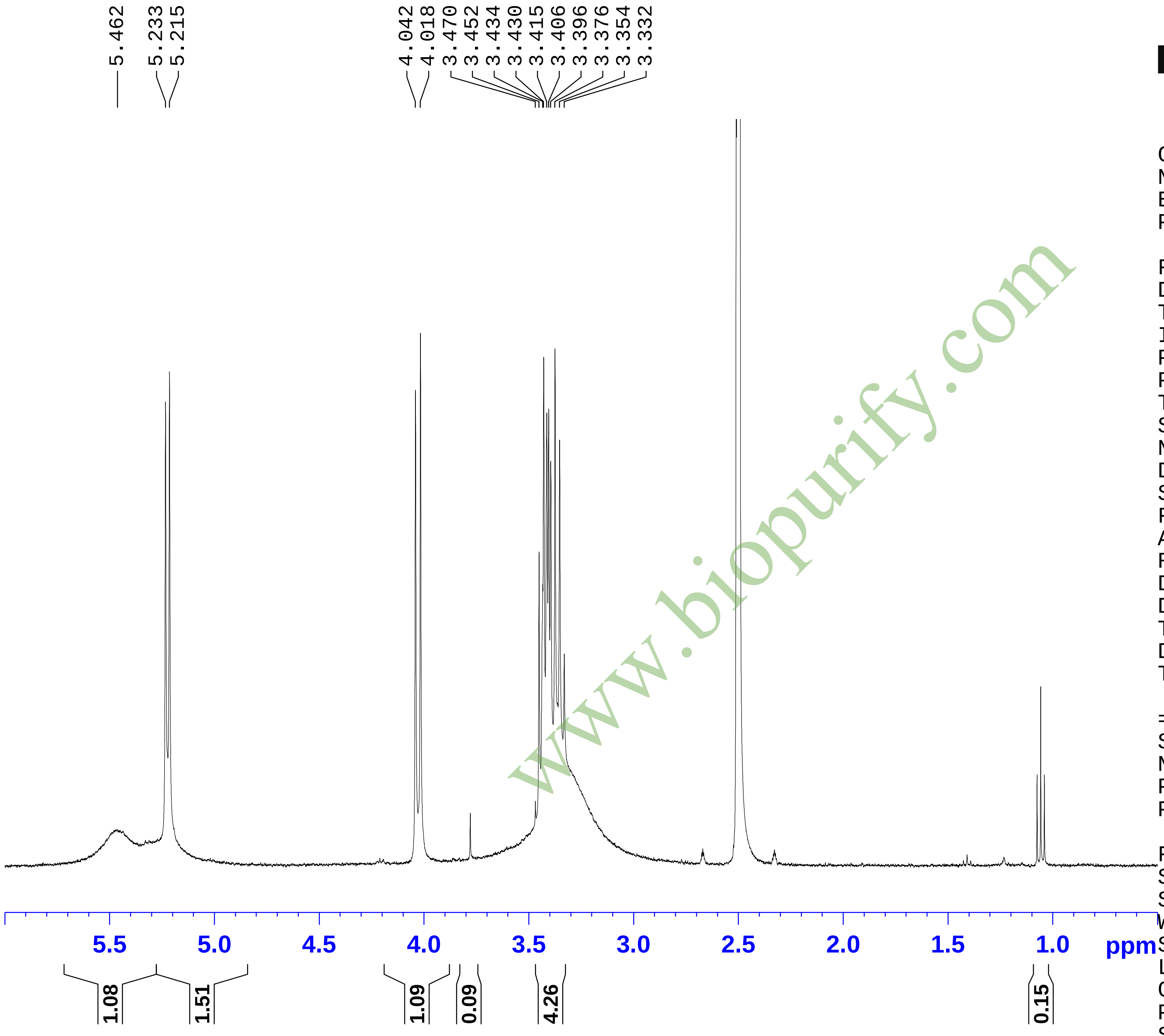

www.biopurify.com

Current Data Parameters
NAME Baicalin-1H NMR DMSO
EXPNO 1
PROCNO 1

F2 - Acquisition Parameters
Date_ 20170225
Time 17.16
INSTRUM spect
PROBHD 5 mm PABBO BB/
PULPROG zg30
TD 65536
SOLVENT DMSO
NS 16
DS 2
SWH 8012.820 Hz
FIDRES 0.122266 Hz
AQ 4.0894465 sec
RG 202.5
DW 62.400 usec
DE 6.50 usec
TE 301.2 K
D1 2.00000000 sec
TD0 1

======== CHANNEL f1 ========
SFO1 400.1324710 MHz
NUC1 1H
P1 14.79 usec
PLW1 11.50800037 W

F2 - Processing parameters
SI 65536
SF 400.1300023 MHz
WDW EM
SSB 0
LB 0.30 Hz
GB 0
PC 1.00
SR 2.34 Hz

SAMPLE INFORMATION

Sample Name:	Baicalein PRF8020642	Acquired By:	System
Sample Type:	Unknown	Sample Set Name:	
Vial:	6	Acq. Method Set:	Baicalein
Injection #:	1	Processing Method:	Samples
Injection Volume:	10.00 ul	Channel Name:	275.0nm
Run Time:	25.0 Minutes	Proc. Chnl. Descr.:	PDA 275.0 nm
Date Acquired:	2017-2-6 11:26:21 CST		
Date Processed:	2017-2-6 12:48:09 CST		

Auto-Scaled Chromatogram

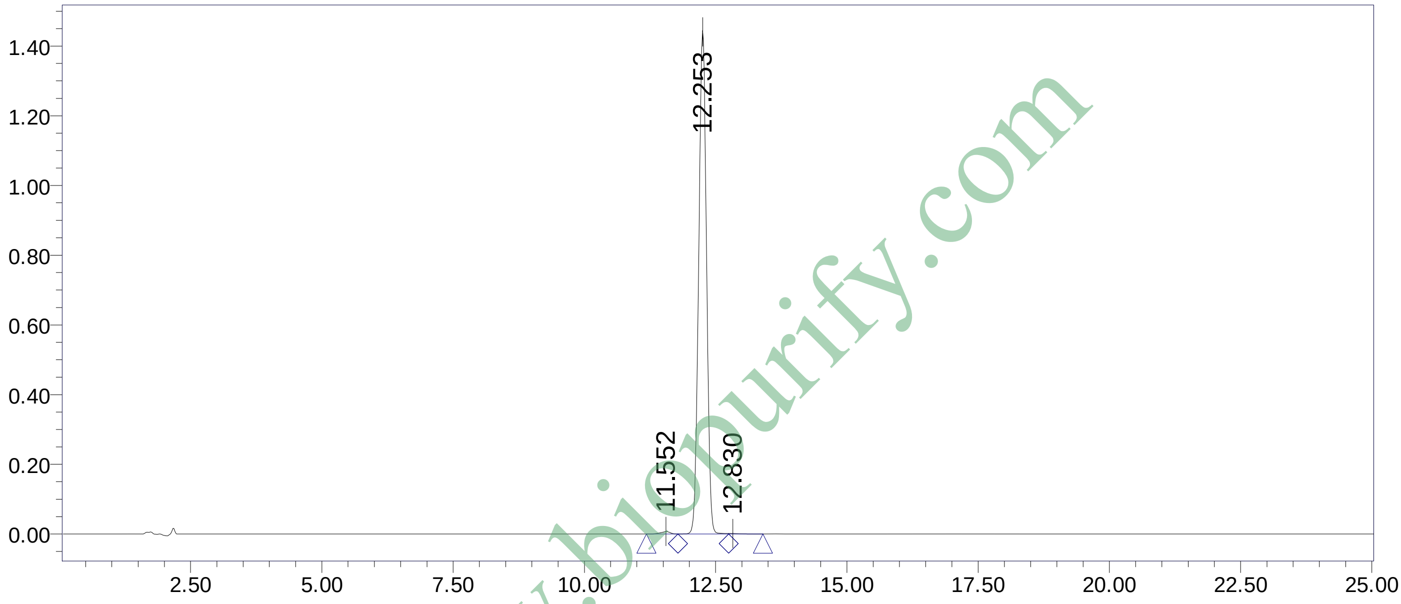

www.biopurify.com

Peak Results

	RT	Area	% Area	USP Plate Count	USP Resolution
1	11.552	88943	0.59	20288.60	
2	12.253	14956771	99.30	31433.73	2.25
3	12.830	15751	0.10		
