# Supplementary figures and images for: Protective Mechanism of the Antioxidant Baicalein toward Hydroxyl Radical-Treated Bone Marrow-Derived Mesenchymal Stem Cells
Source: Molecules. 2018 Jan 20;23(1):223. doi: 10.3390/molecules23010223 (PMC6017293; doi:10.3390/molecules23010223)

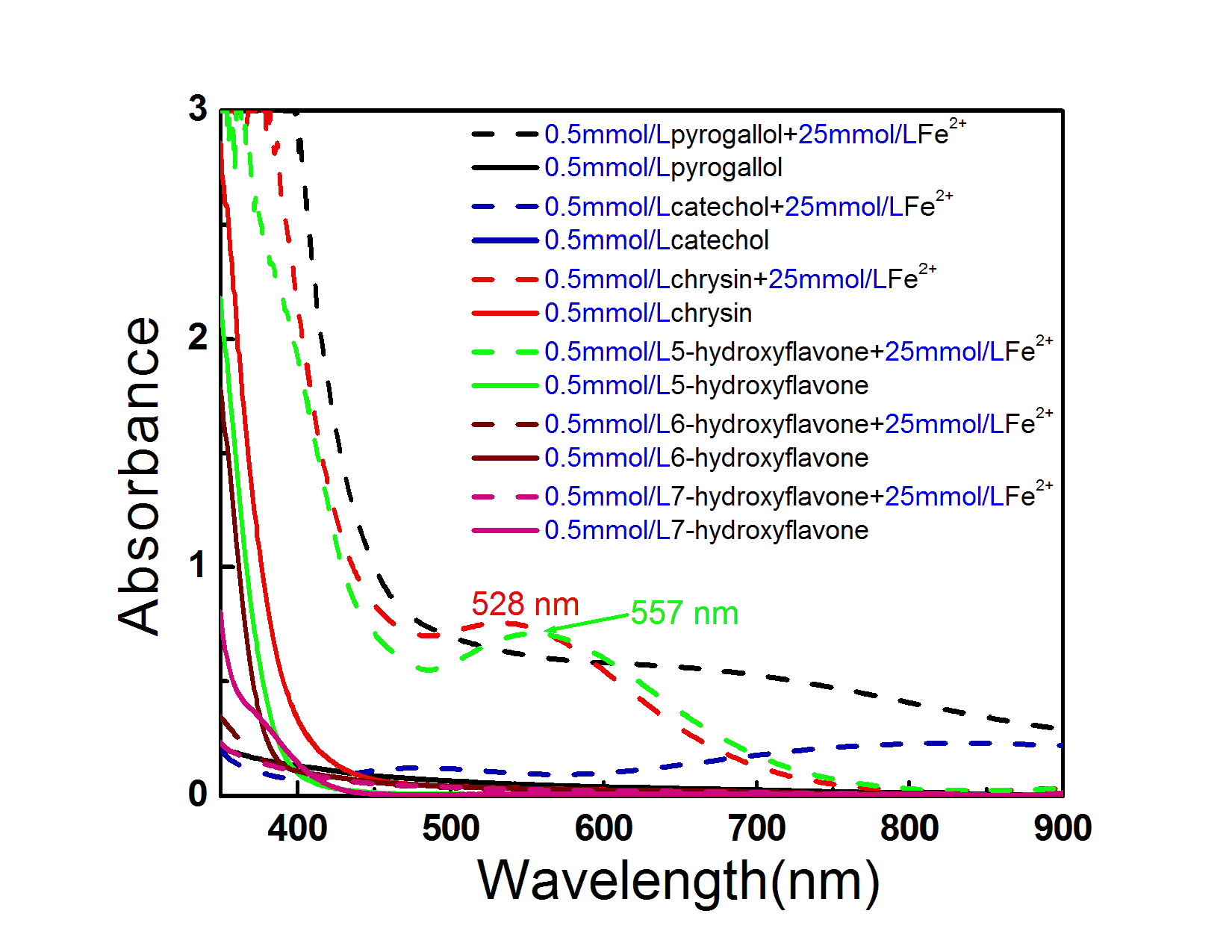

Supplement: Supplementary file 1 [file molecules-23-00223-s001.zip › Suppl.s/Suppl. -1.JPG]

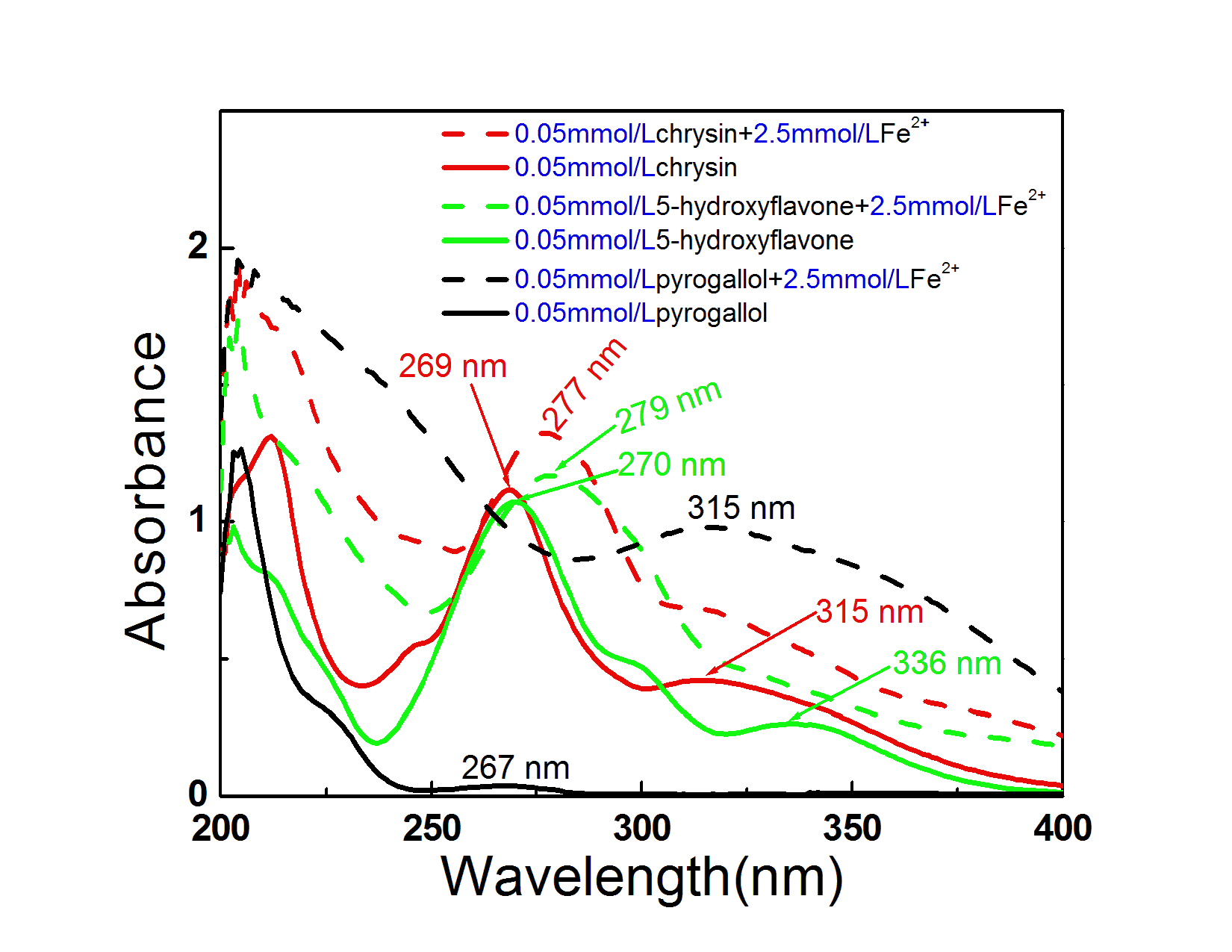

Supplement: Supplementary file 1 [file molecules-23-00223-s001.zip › Suppl.s/suppl. -2.JPG]

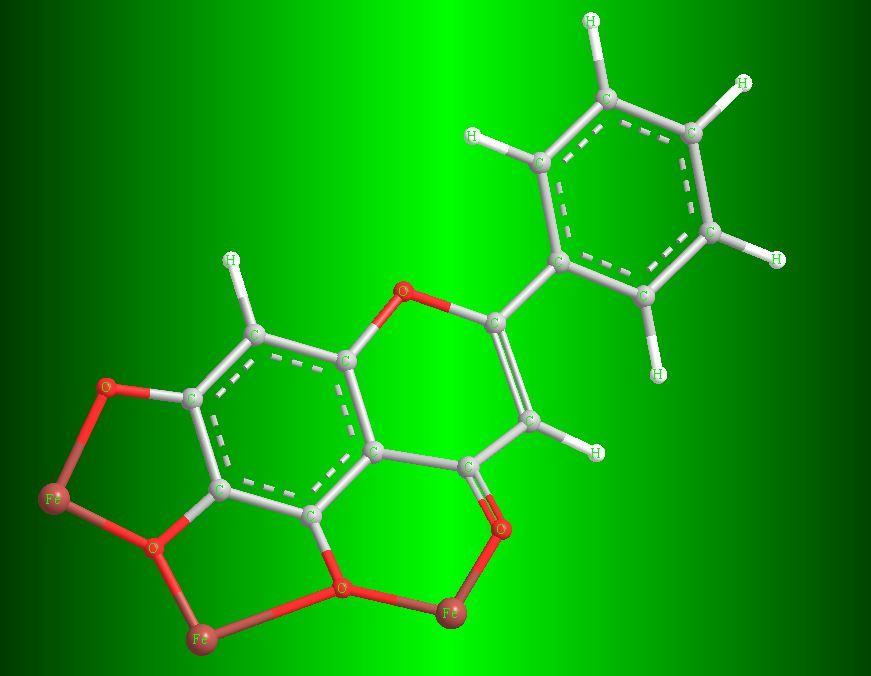

Supplement: Supplementary file 1 [file molecules-23-00223-s001.zip › Suppl.s/Suppl. -3 Fe-complex2.JPG]
